# Circadian rhythms and non-coding RNAs: mechanistic insights, clinical impact, and future opportunities for personalized medicine

**DOI:** 10.1186/s12935-026-04369-1

**Published:** 2026-06-17

**Authors:** Mai A. Abdelmawla, Heba R. Ghaiad, Nora M. Aborehab, Nourhan Elfar, Abdullah F. Radwan, Suzan M. Ismail, Yasmine M. Shahine, Nadia M. Hamdy

**Affiliations:** 1 Biochemistry Department, Faculty of Pharmacy, Cairo University, Kasr el Ainy St, Cairo, 11562 Egypt; 2 Biochemistry Department, Faculty of Pharmacy, Ahram Canadian University, 6 October city, Giza, 12451 Egypt; 3 Biochemistry Department, School of Health and Social Work, University of Hertfordshire hosted by Global Academic Foundation, New Administrative Capital, Cairo, 11578 Egypt; 4 Biochemistry Department, Faculty of Pharmacy, Egyptian Russian University, Badr City, Cairo, 11829 Egypt; 5 Biochemistry Department, College of Pharmacy, University of Kut, Wasit, 52001 Iraq; 6 Pharmacology, Toxicology and Biochemistry Department, Faculty of Pharmacy, Future University in Egypt, New Cairo, Cairo, 11835 Egypt; 7 Microbiology and Immunology Department, Faculty of Pharmacy, Pharos University in Alexandria, Alexandria, Egypt; 8 Biochemistry and Molecular Biology Department, Faculty of Pharmacy, Ain Shams University, Abassia, Cairo, 11566 Egypt

**Keywords:** Circadian rhythm, Clock genes, Chronobiology, Non-coding RNA, microRNA, Chronotherapy

## Abstract

Circadian rhythms are intrinsic 24-hour cycles that regulate diverse physiological processes, including sleep-wake cycles, metabolism, immune function, and hormone secretion. The molecular clockwork is made up of regulatory feedback loops and core clock genes governs these cycles. These rhythms confer adaptive advantages by enabling organisms to anticipate predictable environmental cycles such as light–dark transitions, nutrient availability, and temperature fluctuations, thereby optimizing energy utilization and survival. Non-coding RNAs (ncRNAs), such as microRNAs (miRNAs), long non-coding RNAs (lncRNAs), and circular RNAs (circRNAs), have become important regulators of circadian rhythms on several levels as they alter clock gene transcription. This review provides a comprehensive overview of ncRNAs in circadian regulation, highlighting specific miRNAs that modulate clock genes, as well as lncRNAs that act as scaffolds, sponges, or transcriptional regulators to fine-tune circadian oscillations. Circadian misalignment caused by genetic, environmental, or lifestyle factors disrupts ncRNA expression and has been linked to cancer, cardiovascular disorders, autoimmune conditions, gastrointestinal dysfunction, neurological and psychiatric diseases, and respiratory illnesses. The clinical potential of circulating miRNAs as biomarkers for circadian disruption and the therapeutic approaches targeting ncRNAs to restore circadian balance were discussed, alongside emerging strategies in precision medicine to incorporate interindividual variability into treatment design. Finally, the present review outlined the current challenges and future perspectives to allow better understanding of the ncRNA-circadian interactions, to translate findings into clinical intervention, and to underscore the importance of ncRNAs as both regulators and therapeutic targets in circadian biology and human disease.

## Molecular basis and organization of circadian oscillations

Circadian rhythms are self-sustained, endogenous vacillations within the day that regulate a wide array of physiological, metabolic, and behavioural processes [1]. They are ubiquitous across evolution, from cyanobacteria to humans, reflecting their fundamental role in maintaining organismal homeostasis [2, 3]. These rhythms confer adaptive advantages by enabling organisms to anticipate predictable environmental cycles such as light–dark transitions, nutrient availability, and temperature fluctuations, thereby optimizing energy utilization and survival [4].

**Fig. 1:**
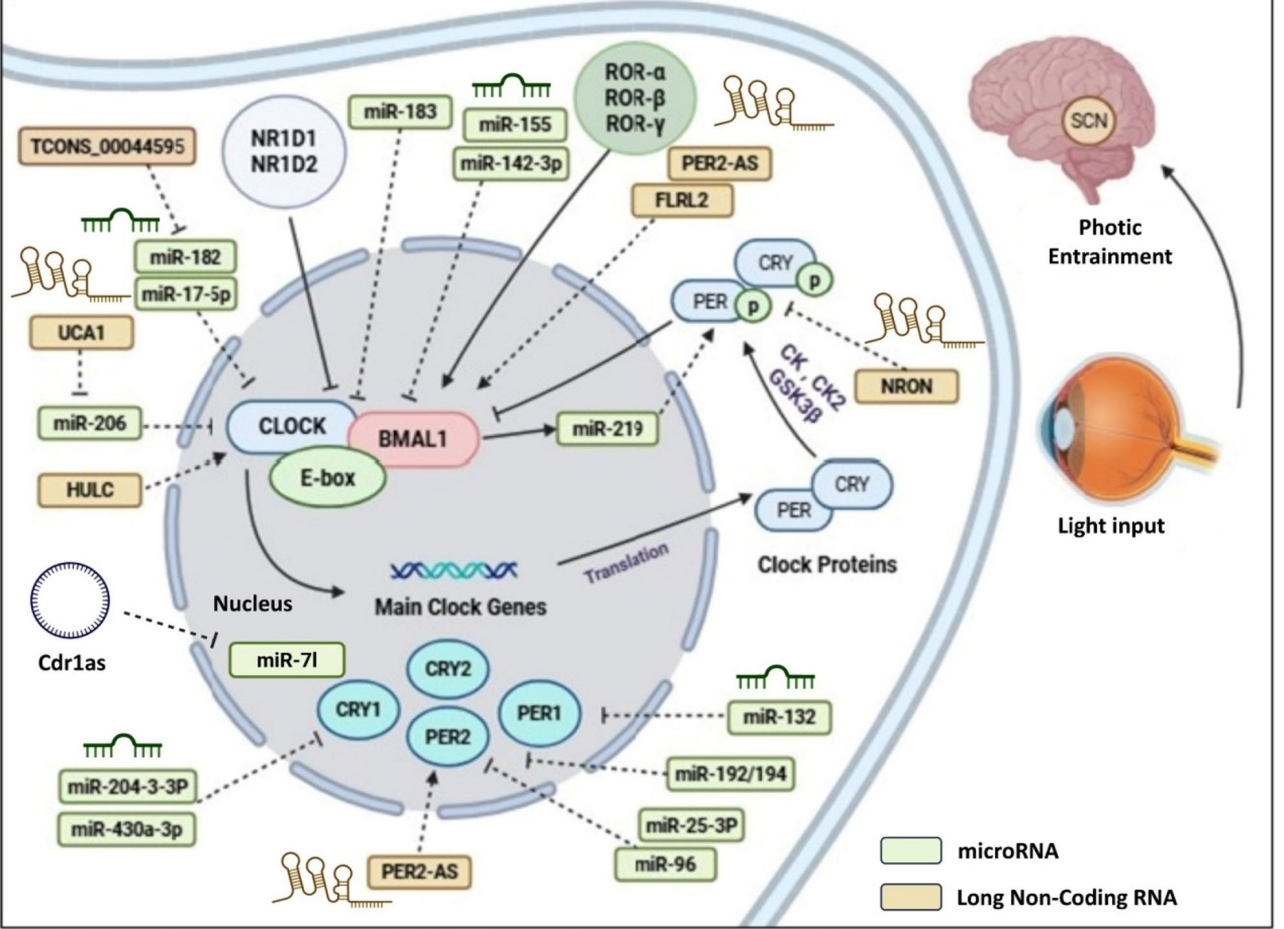
Overview of the core mammalian circadian transcriptional–translational feedback loops. CLOCK and BMAL1 form a heterodimer that binds E-box elements to activate transcription of PER and CRY genes. PER and CRY proteins undergo post-translational modifications, translocate to the nucleus, and hinder CLOCK/BMAL1 activity, thus closing the negative feedback loop. Auxiliary loops involving REV-ERBs (repressors) and RORs (activators) further regulate BMAL1 expression and enhance oscillator stability. Additional post-transcriptional, post-translational, and epigenetic processes fine-tune clock protein abundance and rhythmicity. [BMAL1, Brain and Muscle ARNT-Like 1; CLOCK, Circadian Locomotor Output Cycles Kaput; CRY, Cryptochrome; PER, Period; REV-ERBα/β, Nuclear Receptor Subfamily 1 Group D Members 1 and 2 (NR1D1/NR1D2); RORα/β/γ, Retinoic Acid Receptor-Related Orphan Receptors.]

Circadian timing in mammals is generated by transcriptional–translational feedback loops (TTFLs). In the positive arm of this loop, transcription factor Circadian Locomotor Output Cycles Kaput (CLOCK) form a heterodimer with Brain and Muscle ARNT-Like 1 (BMAL1), then such a heterodimer is attached to E-box enhancer elements to initiate the transcription of Period (PER1-3) and Cryptochrome (CRY1-2), which are main core clock genes as shown in Fig. 1 [5]. The negative limb is formed when the cytoplasmic concentrations of PER and CRY proteins increase; then they undergo post-translational modifications, with subsequent nuclear translocation, where they can inhibit CLOCK/BMAL1 activity. This repression decreases the transcription of PER and CRY, and as they degrade, circadian inhibition will be relieved, allowing the cycle to restart [5, 6].

Additional interlocking loops stabilize this core oscillator. The nuclear receptors REV-ERBα, also known as nuclear receptor subfamily 1 group D member 1 (NR1D1) and REV-ERBβ (or NR1D2), act as transcriptional repressors of BMAL1, while retinoic acid receptor-related orphan receptor α (RORα), RORβ, and RORγ function as activators, thereby fine-tuning BMAL1 expression and enhancing rhythmic robustness [7]. Post-transcriptional and post-translational modifications, including acetylation, ubiquitination, and SUMOylation, further regulate the stability, subcellular localization, and activity of clock proteins [8]. Epigenetic machineries, including the rhythmic chromatin remodeling and histone modifications, can also be involved in the temporal control of gene expression [9].

The circadian system is hierarchically organized [10]. The master circadian pacemaker resides in the suprachiasmatic nucleus (SCN) of the anterior hypothalamus, which receives light input from intrinsically photosensitive retinal ganglion cells via the retinohypothalamic tract to align rhythms with the day–night cycle [11]. The SCN synchronizes peripheral clocks located in nearly all tissues, including the heart, liver, adipose tissue, muscle, and immune cells, through neuronal, endocrine, and metabolic signals [12]. Peripheral clocks share the same molecular machinery as the SCN but can also be entrained by non-photic cues, including feeding–fasting cycles, temperature, and physical activity [12].

High-throughput transcriptomic studies indicate that up to half of all protein-coding genes display circadian rhythmicity in at least one mammalian tissue. These rhythmic transcripts encode enzymes, transporters, receptors, and structural proteins that underlie daily cycles of glucose and lipid metabolism, xenobiotic detoxification, cell cycle progression, DNA repair, hormone secretion, and neuronal activity [13, 14]. Thus, the circadian system acts as a temporal coordinator, aligning cellular and systemic functions with environmental demands [15].

## The core machinery and transcriptional control

The circadian clock is an intrinsic timekeeping system found in nearly all organisms that orchestrates approximately 24-hour rhythms in behaviour and physiology [16]. At the molecular level, this clock relies on conserved TTFLs involving core clock genes. The heterodimeric transcription factors CLOCK and BMAL1 play a pivotal role by binding to specific DNA sequences called E-boxes, thereby activating the transcription of genes including PER1, PER2, PER3 and CRY1, CRY2 [17, 18]. The protein products of these genes accumulate, form complexes, and feed back to inhibit the activity of CLOCK: BMAL1 [19], thus repressing their own transcription. This negative feedback loop generates rhythmic oscillations essential for circadian timing [20, 21]. However, this simplified sequence hides a dense regulatory network, such as transcription factors, chromatin remodeling, post-transcriptional modifications (PTMs), non-coding RNAs (ncRNAs), translational control and systemic cues, each sculpt the rhythm in amplitude, period and tissue specificity. Modern work emphasizes that regulation beyond simple transcriptional feedback is necessary for robustness and flexibility of circadian timing.

Complementary regulatory loops add robustness and precision to the clock. Notably, the nuclear receptors REV-ERB and ROR compete to regulate BMAL1 gene expression via retinoic acid-related orphan receptor response elements (ROREs) [19]. REV-ERBs repress while RORs activate BMAL1 transcription [22]. This creates an intricate balance that fine-tunes the timing and amplitude of oscillations, linking the CLOCK to metabolic and physiological processes [23, 24], creating the genomic architecture that converts molecular timing into daily waves of gene expression.

Post-translational modifications are central determinants of circadian period length, phase precision, amplitude, and oscillator robustness. Sequential phosphorylation, ubiquitination, acetylation, SUMOylation, and controlled proteolysis regulate the abundance, complex assembly, nuclear entry and exit, transcriptional repressor activity, and degradation of PER, CRY, BMAL1, and CLOCK proteins [25–27]. Therefore, it is important not to refer generically to “casein kinases,” because the circadian roles of casein kinase 1 isoforms are functionally distinct. CK1δ, encoded by CSNK1D, and CK1ε, encoded by CSNK1E, phosphorylate PER proteins in temporally ordered patterns, thereby shaping PER/CRY turnover, nuclear trafficking, and circadian period length [25–27]. Genetic and pharmacological studies indicate that CK1δ has a dominant role in maintaining the near-24-hour mammalian clock, whereas CK1ε contributes in a more context-dependent and partially compensatory manner [28, 29]. This isoform specificity is essential because altered PER phosphorylation kinetics can shift the timing of PER accumulation, nuclear repression of CLOCK–BMAL1, and subsequent proteasomal degradation, ultimately changing circadian period and phase [30].

At the mechanistic level, CK1δ/CK1ε-mediated phosphorylation of PER2 operates as a phosphorylation-dependent timing module, often described as a PER2 “phosphor-switch” [30]. Phosphorylation at stabilizing sites delays PER2 degradation and supports the temporal delay required for negative feedback, whereas phosphorylation at phosphor-degron regions promotes recruitment of ubiquitin-dependent degradation machinery [30]. Thus, the balance between stabilizing and destabilizing phosphorylation events determines whether PER2 persists long enough to form an effective PER/CRY repressor complex or is rapidly degraded. This phosphorylation–ubiquitination coupling is reinforced by E3 ubiquitin ligases and deubiquitinases that tune clock protein half-lives [31]. For example, FBXL3 and FBXL21 exert compartment-specific and opposing effects on CRY stability, with FBXL3 promoting CRY degradation and FBXL21 modulating CRY turnover in a manner that influences circadian period length [31]. Together, CK1δ/CK1ε-dependent PER phosphorylation and ubiquitin-mediated PER/CRY proteolysis generate the biochemical delay required for a stable 24-hour oscillator [25, 30, 31].

Within this framework, ncRNAs can interface with PTM-controlled circadian timing through several mechanistic layers. First, miRNAs and lncRNAs may alter the abundance of PER, CRY, BMAL1, CLOCK, or kinase-associated regulatory components, thereby changing the amount of substrate available for CK1δ/CK1ε-mediated phosphorylation and subsequent degradation [32–34]. Second, ncRNAs may influence ubiquitin-dependent clock protein turnover by regulating pathways that affect E3 ligases, deubiquitinases, or proteasomal activity, thereby shifting PER/CRY half-lives and feedback timing. Third, lncRNAs can act as molecular scaffolds that organize PTM, nuclear transport, and degradation machinery. A strong example is NRON, which forms a ribonucleoprotein complex containing kinase-, phosphatase-, proteasome-, and nuclear transport-associated proteins. This complex regulates PER/CRY nucleocytoplasmic trafficking, alters circadian amplitude and period, and links ncRNA scaffolding directly to phosphorylation-dependent clock control [35].

In addition to scaffolding PTM-related protein complexes, circadian lncRNAs may influence oscillator robustness through chromatin-level regulation. Enhancer-associated circadian lncRNAs, including lnc-Crot, mark regulatory regions bound by circadian transcription factors such as BMAL1 and REV-ERBα and participate in long-range enhancer–promoter organization [36]. Although this mechanism does not directly phosphorylate PER or CRY proteins, it can indirectly modify feedback strength by shaping the rhythmic transcriptional availability of clock-controlled genes and regulatory factors [36, 37]. Therefore, ncRNAs should be viewed not only as post-transcriptional regulators of clock gene mRNAs, but also as higher-order modulators of PTM-dependent timing, protein stability, nuclear trafficking, chromatin architecture, and oscillatory buffering capacity.

Epigenetic mechanisms provide a crucial layer of regulation that fine-tunes physiological and pathological mechanisms [38–41]. CLOCK, beyond acting as a transcription factor, has intrinsic histone acetyltransferase activity that acetylates histone H3 (e.g., H3K14ac), facilitating open chromatin states, while the NAD⁺-dependent deacetylase SIRT1 counterbalances this action and directly links circadian regulation to cellular metabolic status [42, 43]. Oscillations in histone methylation also play a role: the methyltransferase MLL1 promotes activating H3K4me3 at CLOCK: BMAL1 targets, whereas EZH2-mediated H3K27me3 provides a repressive counterpart, creating rhythmic patterns of chromatin accessibility [44, 45]. ATP-dependent chromatin remodelers such as SWI/SNF further reposition nucleosomes in a time-of-day-dependent manner, modulating transcriptional amplitude [46, 47]. DNA methylation adds longer-term stability to circadian regulation, potentially reinforcing rhythms in response to chronic cues such as light or feeding [43, 48]. Furthermore, ncRNAs, including lncRNAs and enhancer RNAs, add another epigenetic dimension by guiding chromatin modifiers to clock gene loci and facilitating long-range enhancer/promoter interactions, with lnc-UC regulating REV-ERBs as an example [49–51]. Collectively, these epigenetic processes-histone modifications, DNA methylation, chromatin remodeling and ncRNA activity- cooperate with the canonical transcriptional-translational feedback loop to ensure the robustness, plasticity, and environmental responsiveness of circadian rhythms.

## Organ-specific circadian regulation in mammalian physiology

**Fig. 2:**
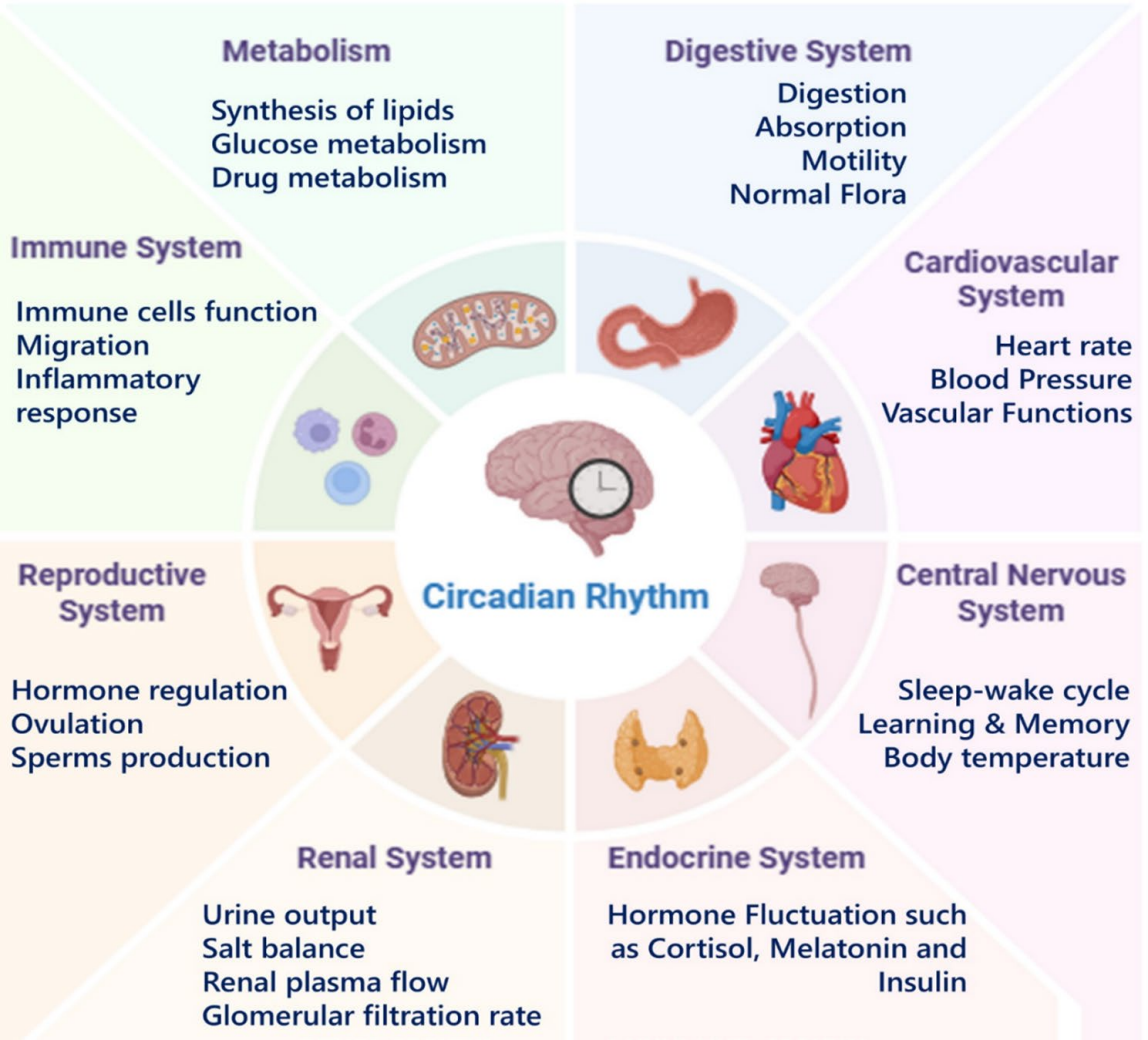
Organ-specific roles of the circadian rhythm in physiological regulation. The circadian system, organized by the brain’s central clock, synchronizes peripheral clocks of the major organs. It harmonized sleep-wake cycles, cognition, cardiovascular function, metabolic function in the liver, renal filtration and electrolyte balance, endocrine hormone secretion, and skeletal muscle metabolism and performance. **[**SCN, Suprachiasmatic Nucleus; BMAL1, Brain and Muscle ARNT-Like 1; PER, Period; RAAS, Renin–Angiotensin–Aldosterone System.]

Circadian rhythms coordinate tissue-specific physiological and metabolic processes across nearly all organs in the body. Although peripheral tissues possess autonomous molecular clocks, these oscillators remain synchronized through hierarchical regulation by the suprachiasmatic nucleus (SCN), which integrates environmental light cues with systemic neuroendocrine and metabolic signaling. Organ-specific circadian programes regulate diverse processes, and any disruption of circadian coordination contributes to the pathogenesis of metabolic, cardiovascular, inflammatory, and neurodegenerative disorders, highlighting the importance of temporal regulation in maintaining physiological homeostasis, as shown in Fig. 2.

The central circadian pacemaker located in the SCN of the anterior hypothalamus synchronizes peripheral oscillators throughout the body and is believed to regulate behavioural and physiological rhythmicity. Through neuronal and hormonal outputs, the SCN coordinates sleep-wake cycles, body temperature, feeding behaviour, hormone secretion, and autonomic nervous system activity [11]. Circadian regulation also plays a critical role in higher brain functions, including cognition, attention, memory consolidation, and synaptic plasticity. Disruption of neural circadian signaling has been associated with impaired cognitive performance, mood disorders, neuroinflammation, and neurodegenerative diseases [52].

Because of possible implications for patient care, research on circadian rhythms in the cardiovascular system is becoming increasingly significant. Circadian regulation is essential for cardiovascular physiology and influences heart rate, vascular tone, endothelial function, blood pressure, thrombosis, and myocardial metabolism. Several cardiovascular events, including myocardial infarction and stroke, display strong circadian patterns, with increased incidence during the early morning hours [53]. Core clock genes such as BMAL1 and PER2 regulate endothelial integrity, inflammatory signaling, lipid metabolism, oxidative stress responses, and vascular homeostasis. Circadian disruption may therefore contribute to hypertension, endothelial dysfunction, vascular inflammation, and increased cardiovascular risk [54].

The liver represents one of the most metabolically active peripheral circadian oscillators and integrates feeding–fasting cycles with transcriptional regulation of glucose metabolism, lipid synthesis, bile acid homeostasis, mitochondrial energetics, and xenobiotic detoxification pathways [55]. Hepatic clock genes rhythmically regulate enzymes involved in glycolysis, gluconeogenesis, fatty acid metabolism, and cholesterol biosynthesis, thereby coordinating metabolic activity with nutrient availability. Dysregulation of hepatic circadian rhythms has been implicated in insulin resistance, obesity, metabolic syndrome, and the progression of fatty liver disease [56].

In the kidney, circadian clocks regulate multiple aspects of renal physiology and metabolism, including glomerular filtration rate, electrolyte transport, tubular reabsorption, urine concentration, and blood pressure homeostasis. Renal clock genes rhythmically control the expression and activity of sodium transporters, ion channels, and components of the renin–angiotensin–aldosterone system (RAAS), thereby coordinating daily fluctuations in sodium balance and fluid homeostasis [57]. Emerging evidence also indicates that circadian regulation influences renal mitochondrial metabolism, oxidative stress responses, and oxygen utilization within tubular epithelial cells. Disruption of renal circadian rhythms has been associated with hypertension, chronic kidney disease, altered electrolyte handling, and impaired metabolic homeostasis. Importantly, several renal physiological processes, including glomerular filtration and tubular transport activity, exhibit peak activity during the active phase and decline during the resting phase, highlighting the temporal organization of kidney function [58].

Additionally, one important systemic regulator of homeostatic processes is hormones. Endocrine systems exhibit pronounced circadian rhythmicity that contributes to the temporal coordination of systemic physiology and metabolic adaptation. Hormones including cortisol, melatonin, growth hormone, insulin, and glucocorticoids display oscillatory secretion patterns that synchronize metabolism, immune responses, stress adaptation, and sleep–wake behaviour with environmental cycles. Circadian disruption within endocrine networks may impair hormonal homeostasis and contribute to metabolic disorders, immune dysregulation, reproductive abnormalities, and altered stress responses [59].

About 45% of the body’s mass is made up of skeletal muscle, making it one of the biggest organs. The circadian expression of around 2300 genes in skeletal muscle contributes to a variety of processes, such as transcription, metabolism, and myogenesis. Skeletal muscle contains an intrinsic circadian clock that regulates transcriptional programes involved in energy metabolism, mitochondrial function, myogenesis, protein turnover, and contractile performance. Circadian oscillations in skeletal muscle are entrained not only by central SCN signaling but also by feeding behaviour and physical activity. Experimental circadian disruption has been associated with altered fibre composition, impaired mitochondrial respiration, reduced insulin sensitivity, and decreased muscle performance, highlighting the importance of temporal regulation in musculoskeletal physiology [60].

Collectively, organ-specific circadian clocks coordinate metabolic, endocrine, cardiovascular, renal, and neuromuscular physiology through temporally regulated gene expression and inter-organ signalling pathways, thereby preserving systemic homeostasis and adaptive responses to environmental cues.

## Role of ncRNAs in circadian regulation

**Fig. 3:**
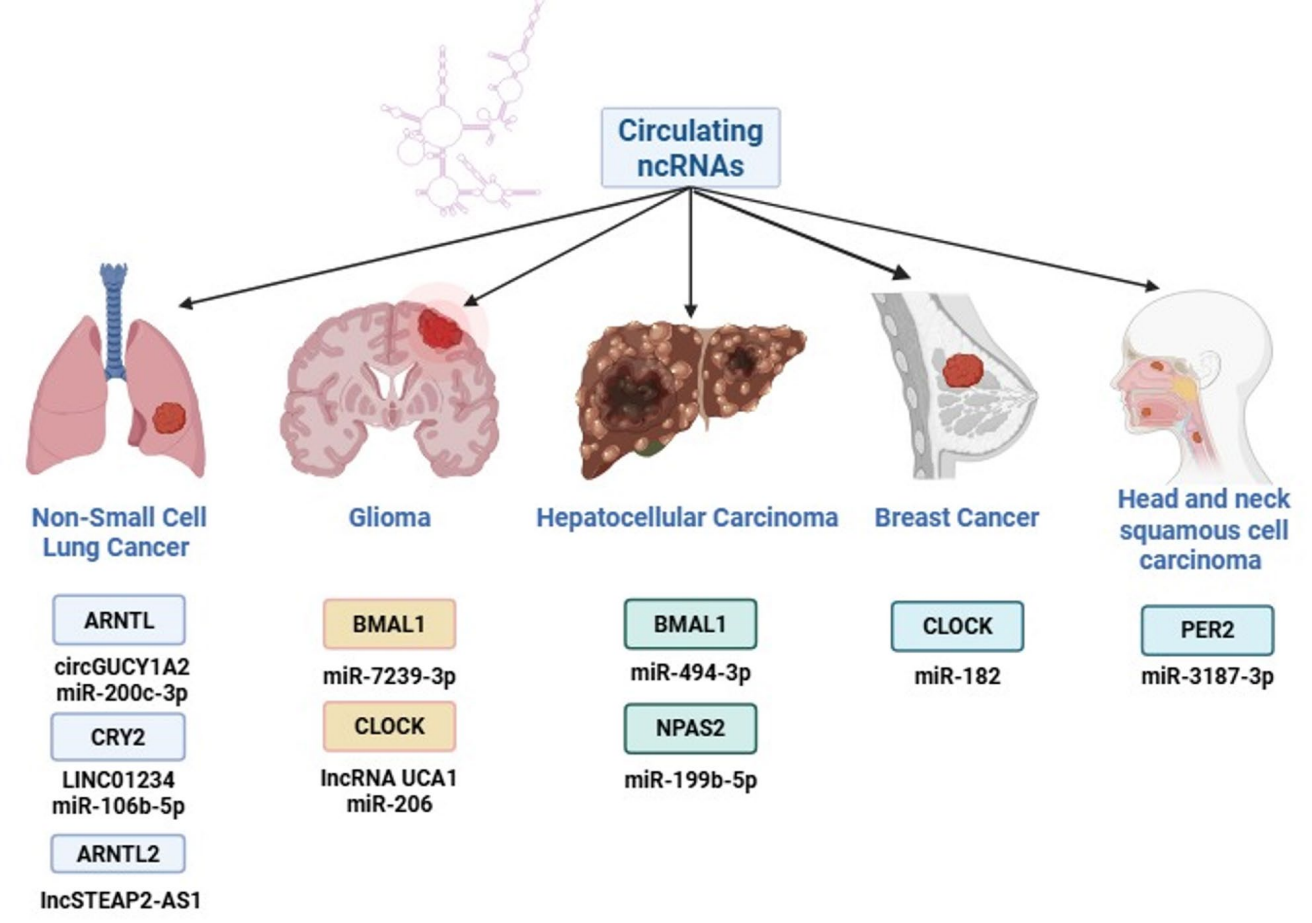
Circulating non-coding RNAs associated with circadian clock dysregulation in different cancer types. Circulating ncRNAs involved in circadian rhythm interruption are connected to numerous malignancies. In non-small cell lung cancer, dysregulated ARNTL, CRY2, ARNTL2, circGUYC1A2, LINC01234, miR-200c-3p, miR-106b-5p, and lncSTEAP2-AS1 have been reported. Glioma is associated with altered BMAL1, CLOCK, miR-7239-3p, lncRNA UCA1, and miR-206. In hepatocellular carcinoma, BMAL1, NPAS2, miR-494-3p, and miR-199b-5p show circadian-linked changes. Breast cancer exhibits CLOCK and miR-182 dysregulation, while head and neck squamous cell carcinoma is concomitant with altered PER2 and miR-3187-3p. [ARNTL/BMAL1, Aryl Hydrocarbon Receptor Nuclear Translocator-Like 1; CLOCK, Circadian Locomotor Output Cycles Kaput; CRY2, Cryptochrome 2; PER2, Period 2; NPAS2, Neuronal PAS Domain Protein 2; ncRNAs, Non-coding RNAs; lncRNA, Long Non-coding RNA; circRNA, Circular RNA; miR, MicroRNA.]

The ncRNAs, including miRNAs, lncRNAs, and circRNAs have emerged as important regulators of circadian rhythms through transcriptional, post-transcriptional, epigenetic, and translational mechanisms (Fig. 3) [61]. miRNAs are short non-coding transcripts that commonly regulate gene expression through mRNA degradation or translational repression, whereas lncRNAs and circRNAs can modulate chromatin organization, transcription factor activity, RNA stability, protein interactions, and competing endogenous RNA (ceRNA) networks [62, 63]. However, these ncRNA subclasses should not be viewed as mechanistically isolated categories, as considerable functional overlap exists among them [64, 65]. Depending on the biological context, miRNAs, lncRNAs, and circRNAs may act as molecular guides, scaffolds, decoys, or sponges that collectively fine-tune the expression of several genes [66–68]. Through these interconnected regulatory mechanisms, ncRNAs influence the amplitude, phase, robustness, and tissue specificity of circadian oscillations, while their dysregulation has been implicated in circadian-related disorders including cancer, metabolic syndrome, inflammatory diseases, and sleep dysfunction [69, 70].

Among these, miRNAs have been most extensively characterized for their post-transcriptional control of circadian gene networks [71]. Many miRNAs display rhythmic expression and directly regulate core oscillator components [72, 73]. For instance, miR-375 has been identified as a key post-transcriptional regulator of circadian rhythm through its direct interaction with the clock gene timeless [74]. The miR-375 oscillates in pacemaker neurons and binds to the TIM 3′UTR, reducing TIM protein levels. Overexpression of miR-375 caused arrhythmic locomotor activity and disrupted sleep, while its inhibition lengthened the circadian period. These findings establish miR-375 as a neuron-specific regulator linking molecular oscillators with circadian behaviour and sleep homeostasis [74].

Another example, miR-397 regulates the circadian rhythm in plants by mediating a feedback loop with core clock genes. A recent study showed that a natural variant of MIR397a in rice alters circadian timing by rhythmically repressing OsLAC genes, which in turn affect OsPRR37, a key regulator of photoperiodic flowering and circadian rhythm. Plants carrying this variant displayed changes in circadian rhythmicity, demonstrating how miR397 contributes to clock control and natural variation in circadian regulation [75]. A previous study revealed that miR-17-5p regulates the circadian rhythm by modulating the expression of core clock components. It demonstrated that miR-17-5p directly targets the 3′UTR of Clock mRNA, thereby reducing CLOCK protein levels and altering circadian dynamics. Overexpression of miR-17-5p lengthened the circadian period and attenuated oscillatory amplitude, whereas inhibition of miR-17-5p increased CLOCK expression and restored more robust rhythms. These results position miR-17-5p as a post-transcriptional regulator within the TTFL, where it fine-tunes CLOCK abundance to maintain the stability and precision of circadian oscillations [76]. Also, miR-219, rhythmically expressed under CLOCK–BMAL1 control in the SCN, shortens the circadian period by promoting timely gene expression. A previous study showed that knockdown of miR-219 delays PER induction, slowing progression through the TTFL and lengthening the period by around 10–20 min [77].

Conversely, normal miR-219 activity advances PER accumulation, accelerating feedback repression and maintaining a shorter, more precise circadian cycle [78]. Computational modelling by Liu & Wang (2012) further confirmed that miR-219 stabilizes oscillations by enhancing the speed and robustness of TTFL dynamics [77]. In contrast, miR-132 is primarily regulated by photic entrainment cues rather than direct clock oscillation. Light stimulation induces miR-132 expression in the SCN through the MAPK/CREB signaling pathway in a phase-dependent manner, particularly during the subjective night. In turn, miR-132 regulates circadian entrainment by dampening light-induced phase shifts. It demonstrated that light pulses elevate miR-132 expression in the SCN, which in turn attenuates CREB-dependent PER1 induction. This negative feedback reduces the magnitude of phase resetting, thereby preventing excessive or unstable entrainment to environmental light cues [78]. Another study further showed through computational modelling that miR-132 enhances circadian robustness by buffering the clock against strong light-induced perturbations [77]. Furthermore, multiple lncRNAs have been identified as BMAL1- or REV-ERBα-regulated transcripts, including ADIRF-AS1 and enhancer-associated hepatic lncRNAs [36, 79]. Together, these findings demonstrate that ncRNAs not only regulate circadian clock function but are also temporally controlled by circadian and light-responsive signalling pathways.

Another recent study showed that the miR-183/96/182 cluster plays a significant role in shaping circadian rhythms, with each member contributing in distinct ways. It revealed that miR-183 influences circadian timing in both cell models and living systems; its loss was linked to arrhythmic behaviour in mice and tissue-specific changes such as a shorter retinal period and altered lung phase. Among the cluster, miR-96 has the clearest molecular target, directly repressing PER2 and thereby dampening oscillatory amplitude within the transcriptional–translational feedback loop. miR-182, on the other hand, was predicted to act on CLOCK, though this regulation appeared weaker and context-dependent, becoming more evident in tissue-level effects rather than in direct molecular assays. When the entire cluster was knocked out, heterozygous mice displayed shorter free-running periods, while homozygous animals became completely arrhythmic under constant darkness. Together, these findings illustrate that the miR-183/96/182 cluster operates on multiple levels, directly tuning core clock genes like PER2 while also influencing peripheral clocks in a tissue-specific manner, making it a key modulator of circadian timing [80].

Additionally, light-responsive miRNAs play a pivotal role in fine-tuning circadian regulation. In zebrafish, miR-204-3-3p and miR-430a-3p directly target the 3′UTRs of cry1a and cry1b, respectively, both of which encode cryptochromes that act as core repressors within the transcription–translation feedback loop. By modulating cryptochrome expression levels, these miRNAs adjust the amplitude and phase of circadian oscillations in response to environmental light cues. Experimental manipulation of miR-204-3-3p or miR-430a-3p altered the expression profiles of key clock components, including CLOCK1a, BMAL1b, PER1b, PER2, and PER3, and led to measurable disruptions in locomotor activity rhythms. Collectively, these findings demonstrate that miR-204-3-3p and miR-430a-3p act as post-transcriptional regulators that link light input to both molecular and behavioural circadian outputs in zebrafish [71].

Collectively, these findings indicate that circadian-associated miRNAs function primarily as dynamic post-transcriptional modulators that buffer clock gene expression against environmental and physiological perturbations. Although individual miRNAs target distinct clock components, several recurring mechanistic themes emerge, including regulation of PER/CRY accumulation, modulation of CLOCK–BMAL1 activity, and fine-tuning of light-induced entrainment pathways. Importantly, many circadian miRNAs appear to regulate oscillator robustness and phase precision rather than acting as simple on/off switches of clock activity, suggesting that miRNA networks contribute to the adaptive flexibility and stability of circadian timing systems.

In addition to microRNAs, lncRNAs regulate circadian rhythms by modulating transcription, chromatin state, and RNA stability. Unlike microRNAs, which act mainly post-transcriptionally, lncRNAs function as scaffolds, decoys, or antisense transcripts at clock gene loci [81]. For instance, a recent study revealed that LncRNA-MSTRG.19083.1 regulates the circadian rhythm by modulating the cAMP pathway through a ceRNA mechanism. It showed that this lncRNA acts as a sponge for miR-429-y, preventing repression of NTRK2, a receptor involved in cAMP signaling. Since the cAMP pathway directly influences circadian oscillations, altered expression of MSTRG.19083.1 in yak cryptorchidism was linked to disrupted circadian gene expression and impaired testicular function. These results indicate that MSTRG.19083.1 integrates circadian regulation with reproductive physiology by coupling ncRNA networks to intracellular signaling [82]. Earlier research showed that lncRNA RP11-414H17.5 is closely associated with circadian rhythm regulation. It displays rhythmic expression and modulates the activity of core circadian regulators, including BMAL1 and PER2. Its dysregulation disrupts the oscillatory patterns of these genes, leading to impaired circadian control of cellular processes. By directly influencing the TTFL, RP11-414H17.5 contributes to the maintenance of circadian timing and rhythmic stability [83].

Moreover, the lncRNA NRON (non-coding repressor of NFAT) has been implicated in circadian timing by modulating the subcellular localization of clock proteins. NRON interacts with PER2, reducing its phosphorylation and hindering its nuclear entry, thereby lengthening the circadian period and attenuating oscillatory amplitude. By influencing PER2 stability and activity, NRON introduces an additional post-transcriptional checkpoint in the TTFL, linking lncRNA function to clock precision and robustness [35]. Lnc-Crot, a liver-enriched lncRNA, contributes to circadian regulation by mediating higher-order chromatin interactions. It forms chromatin loops that bring distal regulatory regions into proximity with circadian gene loci, thereby coordinating transcriptional output in a time-of-day-dependent manner. In mouse liver, lnc-Crot expression oscillates and participates in the co-regulation of circadian-controlled metabolic pathways, suggesting a role in integrating rhythmic transcription with energy homeostasis [36].

The lncRNAs HULC and UCA1 modulate circadian gene expression by regulating CLOCK mRNA stability. HULC directly stabilizes CLOCK transcripts, enhancing their accumulation and rhythmic expression. In contrast, UCA1 acts indirectly by functioning as a sponge for miR-206, a miRNA that targets CLOCK mRNA. Through these mechanisms, both HULC and UCA1 safeguard CLOCK protein abundance and contribute to the robustness of TTFL oscillations, linking lncRNA-miRNA interactions to circadian timing [81, 84]. In the pineal gland, TCONS_00044595 has been identified as a circadian regulator that enhances CLOCK expression in a miRNA-dependent manner. This lncRNA acts as a competing endogenous RNA (ceRNA) by sequestering miR-182, thereby relieving repression of CLOCK mRNA. By stabilizing CLOCK expression, TCONS_00044595 supports the molecular machinery underlying circadian oscillations, highlighting how tissue-specific lncRNAs fine-tune clock gene expression in neuroendocrine contexts [85].

Additionally, FLRL2, a liver-expressed rhythmic lncRNA, influences circadian timing by modulating BMAL1 mRNA abundance. Although the precise mechanism remains unclear, oscillatory expression of FLRL2 parallels circadian transcriptional patterns in the liver, suggesting that it may act through RNA–RNA or RNA–protein interactions to stabilize BMAL1 levels. By controlling the availability of this core activator, FLRL2 contributes to the maintenance of rhythmic gene expression in peripheral metabolic tissues [81, 86].

The antisense transcript PER-AS, transcribed from the PER2 locus, is a well-characterized lncRNA in mammalian circadian regulation. PER2-AS forms sense–antisense RNA duplexes with PER2 mRNA, influencing its stability, nuclear export, and overall expression. Beyond modulating PER2, PER2AS has been shown to impact BMAL1 expression, thereby extending its influence across both arms of the TTFL. Notably, Per2AS itself is rhythmically expressed in an antiphasic manner relative to Per2 mRNA, indicating that it is integrated within the circadian regulatory network. Per2AS exemplifies how antisense lncRNAs contribute to the fine-tuning of circadian amplitude and robustness [87].

In the fungal clock model Neurospora crassa, the antisense lncRNA qrf is transcribed antiphasic to the core clock gene frq. qrf negatively regulates frq transcription via transcriptional interference and epigenetic modulation, thereby shaping both the amplitude of circadian oscillations and the organism’s responsiveness to light-induced phase shifts. The conservation of antisense-based regulation at circadian loci across species, from qrf in fungi to Per2AS in mammals, underscores the evolutionary importance of lncRNA-mediated control in circadian systems [88].

In contrast to the predominantly post-transcriptional actions of many miRNAs, circadian lncRNAs frequently operate through higher-order regulatory mechanisms involving chromatin remodeling, transcriptional coordination, molecular scaffolding, and organization of ribonucleoprotein complexes. Nevertheless, substantial mechanistic overlap exists among ncRNA subclasses, as several lncRNAs also function through ceRNA-mediated miRNA sequestration or modulation of RNA stability. A recurring feature among circadian lncRNAs is their ability to integrate transcriptional regulation with nuclear trafficking, epigenetic control, and oscillator robustness, thereby extending circadian regulation beyond canonical transcriptional–translational feedback loops.

In addition to miRNAs and lncRNAs, circRNA Cdr1as (also known as ciRS-7) has been identified as a key regulator of circadian function in the SCN. Lochhead et al. (2020) showed that Cdr1as exhibits rhythmic expression in the SCN and is dynamically induced by light stimulation, directly linking it to photic entrainment. Functionally, Cdr1as acts as a molecular sponge for miR-7, thereby modulating the availability of this microRNA to target transcripts involved in neuronal signaling and circadian regulation. By integrating light input with microRNA-mediated control, Cdr1as contributes to phase resetting and is suggested to play a role in stabilizing circadian oscillations in the central pacemaker. These findings establish Cdr1as as the first circRNA directly implicated in mammalian circadian entrainment and highlight its role as a noncoding RNA hub connecting environmental cues with the molecular clock [89].

Together, these findings underscore that ncRNAs represent a multilayered regulatory network essential for the precision and adaptability of circadian rhythms. By targeting mRNAs, regulating chromatin, or sequestering miRNAs, they integrate environmental cues with core oscillators to fine-tune rhythmicity. Their tissue-specific expression highlights their role in maintaining temporal homeostasis, while their dysregulation links them to circadian-related diseases and potential therapeutic interventions.

Overall, emerging evidence suggests that ncRNAs regulate circadian biology through interconnected multilayered networks rather than isolated linear pathways **(**Table 1**).** Multiple ncRNA subclasses frequently converge on the same circadian regulators, particularly BMAL1, CLOCK, PER, and CRY, while individual ncRNAs may simultaneously influence transcriptional regulation, RNA stability, protein localization, chromatin accessibility, and signal transduction pathways. This systems-level organization likely contributes to the robustness, adaptability, and tissue specificity of circadian oscillations under physiological and pathological conditions. Importantly, many currently identified ncRNA-clock interactions appear to converge on broader biological hallmarks including metabolic regulation, inflammatory signaling, stress adaptation, and cellular homeostasis, suggesting that ncRNAs function as integrative regulators linking circadian timing with complex physiological networks.

**Table 1:** Key ncRNAs regulating core circadian clock genes

ncRNA	Target Circadian Gene(s)	Function or Role	Ref.
miR-375	Timeless mRNA (3′UTR)	Neuron-specific regulator; reduces TIM protein; overexpression causes arrhythmicity and sleep disruption; inhibition lengthens period.	[74]
miR-397	OsLAC genes → OsPRR37	Mediates feedback loop; variant alters circadian timing in plants by affecting OsPRR37 expression.	[75]
miR-17-5p	CLOCK mRNA (3′UTR)	Suppresses CLOCK; overexpression lengthens period and attenuates the amplitude; inhibition restores robust rhythms.	[76]
miR-219	PER genes via TTFLs	Shortens the circadian period by advancing Per induction; knockdown delays Per and lengthens the period; stabilises TTFLs.	[77]
	Regulated by CLOCK: BMAL1	Shortens the circadian period; fine-tunes the timing of the circadian clock	[78, 90]
miR-132	PER1 via MAPK/CREB	Phase-dependently induced by photic circadian cues in the SCN via the MAPK/CREB pathway; attenuates CREB-dependent Per1 induction, dampens light-induced phase shifts, and buffers circadian entrainment.	[77, 78]
	Chromatin remodelling genes	Mediates light-dependent phase resetting by repressing chromatin remodelling genes, aiding entrainment	[90]
ADIRF-AS1	BMAL1/PBAF-associated pathways	BMAL1-regulated circadian lncRNA that modulates PBAF-responsive gene expression and influences circadian-associated transcriptional regulation.	[79]
Enhancer-associated hepatic lncRNAs	REV-ERBα-associated targets	REV-ERBα-regulated hepatic lncRNAs involved in rhythmic enhancer activity and circadian transcriptional control in the liver.	[36]
miR-204-3-3p	cry1a (cryptochrome)	Fine-tunes cryptochrome expression in zebrafish, adjusting the amplitude/phase of oscillations; influences molecular clock genes and locomotor activity.	[71]
miR-430a-3p	cry1b (cryptochrome)	Regulates cry1b in zebrafish, altering clock gene expression and behavioural rhythms	[71]
miR-192/194	PER gene family	Inhibits PER genes, leading to a shortening of the circadian period	[91, 92]
miR-122	Predicted core clock genes	Exhibits circadian oscillatory expression in the liver under clock-controlled regulation and contributes to post-transcriptional modulation of circadian metabolic gene networks.	[93, 94]
miR-25-3p	PER2	Post-transcriptional downregulation of PER2 expression	[95]
miR-96	PER2	Modulates circadian period length and amplitude	[96]
miR-142-3p	BMAL1	Binds BMAL1 mRNA 3′UTR, reducing its expression and altering circadian rhythmicity	[97]
miR-155	BMAL1	Represses BMAL1 in macrophages; links circadian regulation to innate immunity and inflammatory responses	[98]
Lnc-UC	REV-ERBα	Modulates REV-ERBα expression via recruitment of NF-κB-associated chromatin modifiers; links circadian and inflammatory pathways	[49]
NRON	PER2	Regulates PER2 phosphorylation and nuclear translocation, affecting circadian period length and amplitude	[35]
Lnc-Crot	Circadian transcription factors	Acts as a chromatin looping hub, orchestrating tissue-specific rhythmic gene expression	[36]
HULC	CLOCK	Stabilises CLOCK mRNA by interacting with its 5′ untranslated region; post-transcriptional regulation	[84]
UCA1	CLOCK (indirect via miR-206)	Serves as a molecular sponge for miR-206, thereby promoting CLOCK expression	[99]
TCONS_00044595	miR-182 / CLOCK mRNA	Enhances CLOCK expression by sponging miR-182 in the pineal gland; supports circadian oscillations	[85]
FLRL2	BMAL1 mRNA	Modulates BMAL1 abundance in mouse liver; the mechanism remains unclear but likely stabilises rhythmic transcription	[81, 86]
PER2AS	PER2 mRNA (sense–antisense duplex)	Rhythmically expressed antisense lncRNA that regulates PER2 expression, influences BMAL1 levels, and modulates circadian amplitude and TTFL robustness.	[87]
qrf (Neurospora)	frq gene (antisense regulation)	Antisense transcript repressing frq; regulates circadian amplitude and light-induced phase shifts in Neurospora crassa	[88]
Cdr1as (circRNA)	miR-7 and core clock genes	Sequesters miR-7, regulating neuronal activity; loss leads to upregulation of core clock genes; important for light entrainment	[89, 100]
mbl (circRNA)	Not specified	Exhibits period-dependent expression; deregulated by light exposure; evolutionarily conserved rhythm regulator	[89]

## Crosstalk between circulating ncRNAs and circadian genes in disease development

Numerous studies have demonstrated that circulating ncRNAs are critical mediators linking circadian misalignment to pathological conditions, providing both mechanistic insights and potential biomarkers for disease progression **(**Table 2**).**

### Cancer

The crosstalk between circulating ncRNAs and circadian genes, which function as key modulators of oncogenic pathways, is strongly associated with tumour initiation and progression. Herein, we summarize key studies that highlight these interactions across various types of cancer. Importantly, accumulating evidence suggests that many ncRNA/clock interactions converge on common oncogenic processes, including metabolic reprogramming, epithelial-mesenchymal transition (EMT), stemness maintenance, and resistance to apoptosis, indicating that circadian dysregulation may contribute broadly to tumour progression rather than representing isolated tumour-specific events.

In non-small cell lung cancer (NSCLC), the circadian gene ARNTL suppresses tumour progression by binding to the circGUCY1A2 promoter region and upregulating its transcription. Elevated levels of circGUCY1A2 sequester miR-200c-3p, relieving its inhibitory effect on phosphatase and tensin homolog (PTEN) expression. The restoration of PTEN inhibits phosphatidylinositol 3-Kinase/protein Kinase B (PI3K/AKT) signaling pathway and ultimately reduces cancer cell proliferation and promotes apoptosis [101]. This finding highlights a tumor-suppressive circRNA-mediated circadian axis in which ARNTL indirectly preserves PTEN signaling, suggesting that maintenance of circadian homeostasis may counteract oncogenic PI3K/AKT activation. In contrast, LINC01234 has been found to promote NSCLC cell growth and inhibit apoptosis through the regulation of CRY2 expression. Chen et al. demonstrated that LINC01234 interacts with the RNA-binding protein heterogeneous nuclear ribonucleoprotein A2/B1 (HNRNPA2B1), which recruits DiGeorge syndrome critical region gene 8 (DGCR8) to induce pri-miR-106b processing. The resulting mature miR-106b-5p downregulates CRY2 and elevates c-Myc expression, thereby enhancing NSCLC progression [102]. Notably, this mechanism contrasts with the tumour-suppressive role of the ARNTL/circGUCY1A2 pathway, illustrating how distinct ncRNA–clock interactions may exert opposing biological effects depending on the clock component involved and the downstream proliferative pathways activated. Lnc STEAP2-AS1 has also been identified in NSCLC progression; it binds with ARNTL2 to upregulate aspartyl-tRNA Synthetase 2, Mitochondrial (DARS2) expression, a process that promotes tumor cell proliferation, migration, and invasion [103]. Collectively, these findings indicate that ncRNAs can either suppress or enhance NSCLC progression through differential regulation of ARNTL family members and CRY signalling pathways, underscoring the context-dependent nature of circadian regulation in tumour biology.

A study conducted in 2021 reported that miR-7239-3p is transferred into glioma cells via exosomes, where it suppresses BMAL1 expression. This suppression promotes cell growth and epithelial–mesenchymal transition, which is characterized by elevated N-cadherin and Vimentin and decreased E-cadherin levels, ultimately driving glioma progression [104]. These findings suggest that BMAL1 dysregulation may contribute to pathological cellular plasticity across multiple disease contexts, although the underlying mechanisms appear to be tissue- and disease-specific. Another study revealed that lncRNA UCA1 upregulates CLOCK expression by sponging miR-206, a mechanism that enhances glioma cell growth and invasion [105]. Interestingly, although both studies demonstrate ncRNA-mediated circadian disruption in glioma, they involve distinct molecular mechanisms, namely BMAL1 suppression versus CLOCK activation, indicating that tumor cells may exploit multiple components of the circadian machinery to achieve similar oncogenic outcomes.

Although the precise roles of ncRNAs in regulating circadian genes that influence metabolic reprogramming in cancer cells remain elusive, growing evidence points to their involvement in tumor progression. For example, Yang et al. found that miR-494-3p–mediated BMAL1 downregulation enhances lipid metabolism reprogramming, resulting in hepatocellular carcinoma (HCC) growth and metastasis. Molecularly, BMAL1 cooperates with enhancer of zeste homolog 2 (EZH2) in HCC cells to repress the transcription of glycerol-3-phosphate acyltransferase mitochondrial (GPAM), a crucial enzyme in lipid biosynthesis. This repression suppresses levels of lysophosphatidic acid (LPA), a mediator of oncogenesis that drives HCC progression [106]. These findings support the emerging concept that circadian regulators participate in the control of tumor metabolic reprogramming. Conversely, Yuan et al. reported that miR-199b-5p suppresses HCC progression by targeting the circadian gene NPAS2 and regulating glucose metabolism reprogramming. Inhibition of NPAS2 expression downregulates hypoxia-inducible factor 1-alpha (HIF-1α), which subsequently lowers glycolytic gene expression and promotes mitochondrial biogenesis in HCC cells [107]. Together, these findings demonstrate that ncRNAs can differentially regulate multiple metabolic pathways, including lipid biosynthesis and glycolytic reprogramming, through distinct circadian targets.

It has been found that miR-182 downregulates CLOCK in breast cancer cells by inducing aldehyde dehydrogenase [108], a marker of breast cancer stem-like cells (BCSCs). ALDH-positive BCSCs drive self-renewal, therapy resistance, and tumorigenic potential, thereby maintaining tumor stemness and contributing to relapse and metastasis [109]. This observation extends the oncogenic consequences of circadian disruption beyond proliferation and metabolism to include stemness maintenance and therapeutic resistance, both of which are increasingly recognised as circadian-regulated processes. In head and neck squamous cell carcinoma (HNSCC), miR-3187-3p negatively regulates PER2 expression and subsequently activates the Wnt/β-catenin pathway, inducing cancer cell migration and invasion [110]. These findings support the proposed tumor-suppressive role of PER2 in coordinating circadian regulation with anti-invasive signaling pathways.

Overall, the studies discussed above collectively indicate that recurring ncRNA/clock axes converge on several conserved oncogenic mechanisms, including EMT activation, metabolic rewiring, stemness maintenance, and inflammatory signaling. Importantly, however, the functional consequences of circadian gene modulation appear highly context-dependent, as some ncRNAs suppress tumor growth while others promote malignancy through distinct clock components. These discrepancies may reflect tissue-specific circadian architecture, tumor heterogeneity, differences in circadian sampling time, or dynamic changes in clock gene function during tumor evolution.

### Cardiovascular Diseases (CVDs)

Recent studies have identified lncRNA-AK023617 as a novel circadian regulator in atherosclerosis, as its expression closely correlates with plaque vulnerability. Mechanistically, AK023617 interacts with BMAL1 to activate immunity-related GTPase family M protein 1 (Irgm1) transcription. The activation of Irgm1 drives macrophage necroptosis through the upregulation of p-RIP3 and p-MLKL, ultimately influencing circadian rhythms of plaque stability and promoting the progression of atherosclerosis [111]. Meanwhile, miR-21 has been shown to regulate atherosclerosis by modulating macrophage apoptosis. Knockdown of both miR-21-5p and miR-21-3p abolishes the circadian expression of XIAP-associated factor 1 (XAF1), leading to increased caspase-3 activity that enhances apoptosis, reduces necrotic core size, and thereby attenuates atherosclerosis progression [112]. Collectively, these findings suggest that ncRNA-mediated circadian dysregulation contributes to multiple stages of atherosclerosis progression, including macrophage necroptosis, endothelial dysfunction, and plaque instability. Importantly, BMAL1 emerges as a recurrent target across inflammatory, cardiovascular, and metabolic disorders, supporting the concept that circadian regulators coordinate common pathological pathways across diverse diseases.

NEAT1 is another circadian-regulated lncRNA implicated in atherosclerosis. In human umbilical vein endothelial cells (HUVECs), exposure to trimethylamine N-oxide (TMAO) induces NEAT1 expression. The upregulation of NEAT1 reduces endothelial cell proliferation by increasing Clock and BMAL1 expression while suppressing MAPK signaling pathways. Interestingly, treatment with asparagus extract (AE) restores NEAT1 expression, alleviates circadian disruption of Clock and BMAL1, and enhances MAPK activity, ultimately improving TMAO-impaired HUVEC proliferation and alleviating atherosclerosis progression [113]. Interestingly, the effects of NEAT1 on Clock and BMAL1 expression appear partially paradoxical, as increased clock gene expression was associated with impaired endothelial proliferation under TMAO exposure. This suggests that vascular circadian dysfunction may not solely depend on reduced clock gene expression but may also involve altered rhythmic coordination or compensatory clock activation under cellular stress conditions.

### Inflammatory diseases and others

Lnc-UC has been identified as a circadian and NF-κB-driven lncRNA that links the molecular clock to ulcerative colitis. Both BMAL1 and NF-κB activation upregulate Lnc-UC expression, which in turn triggers a self-protective mechanism by inducing auto-inhibition of NF-κB in colitis. Mechanistically, Lnc-UC interacts with the chromatin regulator Cbx1, reducing its gene-silencing activity at the Rev-erbα promoter and thereby enhancing Rev-erbα expression. Consequently, Rev-erbα inactivates NF-κB and the NLRP3 inflammasome, establishing an anti-inflammatory state and modulating the diurnal rhythmicity of disease severity [114]. Importantly, both Lnc-UC and other circadian inflammatory ncRNAs appear to establish negative-feedback regulatory circuits that restrain excessive NF-κB activation while preserving rhythmic immune homeostasis, suggesting that certain circadian ncRNAs may function as endogenous anti-inflammatory buffering systems.

Platr4 has also been identified as an oscillating and NF-κB-driven lncRNA that integrates circadian regulation with experimental steatohepatitis. Its rhythmic expression is controlled by Rev-erbα and upregulated by NF-κB activation. Like lnc-UC, Platr4 establishes a self-protective mechanism in steatohepatitis by restraining NF-κB-induced inflammation. Mechanistically, Platr4 interacts with Rxrα, blocking the formation of the NF-κB/Rxrα complex at κB sites within the promoters of the inflammasome components Nlrp3 and Asc, thereby repressing their transcription. Ultimately, Platr4 preserves circadian regulation of inflammatory responses and ameliorates the pathological conditions of steatohepatitis [115]. The similarities between Lnc-UC and Platr4 are particularly notable because both ncRNAs are rhythmically regulated, NF-κB responsive, and mechanistically linked to inflammasome suppression. Such convergence strongly supports the existence of conserved ncRNA/clock/inflammatory axes across distinct inflammatory diseases.

In myeloid cells, the proinflammatory miR-155 is induced by lipopolysaccharide (LPS) in a time-dependent manner that inversely correlates with BMAL1 expression. BMAL1 normally protects mice from LPS-induced sepsis by acting as a brake on inflammation through blockade of NF-κB signaling and suppression of miR-155 induction. In contrast, miR-155 directly targets BMAL1, sensitizing mice to sepsis and resulting in increased NF-κB activity and proinflammatory cytokine production. This establishes a reciprocal regulatory loop in which the circadian clock controls miR-155 induction, while miR-155 represses BMAL1 to alter clock function and circadian regulation of inflammation [116]. Among currently identified inflammatory circadian pathways, the miR-155/BMAL1 axis appears to be one of the most consistently reported mechanisms. Beyond sepsis, altered miR-155 expression and BMAL1 dysregulation have also been implicated in chronic inflammatory states and tumor-associated immune dysregulation, suggesting that this pathway may represent an important molecular link between circadian disruption and inflammatory disease progression. However, some studies have reported context-dependent differences in BMAL1 regulation despite elevated inflammatory signaling, indicating that circadian inflammatory responses may differ according to tissue type, disease stage, or temporal sampling conditions. These discrepancies highlight the importance of incorporating time-resolved experimental designs in future circadian inflammation studies.

Intriguingly, the microRNA hsa-let-7f-1-3p was identified as an inducer of intervertebral disc degeneration (IDD) through direct inhibition of BMAL1 expression. Loss of BMAL1 renders nucleus pulposus cells (NPCs) more vulnerable to apoptosis by impairing autophagy. This impairment reduces NPC viability, disrupts extracellular matrix balance, and diminishes resistance to oxidative stress, thereby increasing susceptibility to IDD [117]. Interestingly, the pathological consequences of BMAL1 suppression in IDD resemble those observed in cancer and inflammatory diseases, particularly impaired cellular homeostasis, increased apoptosis, and altered stress responses. This further supports the concept that BMAL1 functions as an important integrator of tissue resilience across multiple pathological conditions.

Regarding age-related bone loss, knockdown of the age-dependent miR-142-3p enhances the osteogenic potential of BMSCs in aged mice by upregulating BMAL1. Elevated BMAL1 suppresses YAP expression independently of Hippo pathway activation, leading to improved bone formation and restoration of circadian-mediated bone homeostasis [118]. Taken together, these findings indicate that ncRNA-mediated circadian dysregulation extends beyond classical inflammatory diseases and contributes broadly to degenerative, metabolic, and age-related disorders. A recurring observation across these conditions is the central involvement of BMAL1-dependent pathways, reinforcing the notion that disruption of circadian homeostasis may represent a unifying mechanism underlying diverse pathological processes.

Collectively, current evidence suggests the existence of common ncRNA circadian regulatory axes across multiple diseases, including cancers, inflammatory disorders, cardiovascular diseases, and degenerative or age-related conditions. Among these, BMAL1-centred pathways appear to be the most frequently disrupted. Multiple ncRNAs, including miR-155, miR-494-3p, miR-7239-3p, hsa-let-7f-1-3p, and miR-142-3p, directly or indirectly modulate BMAL1 regulation across distinct pathological contexts. Despite differences in disease phenotype, these pathways commonly converge on impaired cellular homeostasis, metabolic reprogramming, inflammatory activation, apoptosis, and enhanced disease progression. Similarly, CLOCK-associated pathways involving UCA1, miR-182, and other ncRNAs repeatedly influence proliferation, stemness, and invasive behaviour in several cancers. PER2-associated pathways also demonstrate recurrent tumor-suppressive functions through regulation of Wnt/β-catenin signaling and circadian control of cell migration. Collectively, these observations suggest that ncRNAs regulate circadian biology through interconnected regulatory networks linking circadian disruption with broad pathological hallmarks rather than through isolated disease-specific mechanisms.

**Table 2:** Crosstalk Between Circulating ncRNAs and Circadian Genes in Disease Development

Disease	Genes	ncRNAs	Role	Ref.
**Cancer**				
Non-small cell lung cancer (NSCLC)	ARNTL	circGUCY1A2/ miR-200c-3p	Suppress NSCLC proliferation and promote apoptosis.	[101]
	CRY2	LINC01234/ miR-106b-5p	Promote NSCLC growth and inhibit apoptosis	[102]
	ARNTL2	lnc STEAP2-AS1	Promote NSCLC proliferation, migration, and invasion	[103]
Glioma	BMAL1	miR-7239-3p	Promote glioma cell progression	[104]
	CLOCK	lncRNA UCA1/ miR-206	Promote glioma cell progression	[105]
Hepatocellular carcinoma (HCC)	BMAL1	miR-494-3p	Promote HCC progression	[106]
	NPAS2	miR-199b-5p	Suppress HCC progression	[107]
Breast cancer	CLOCK	miR-182	Promote breast cancer progression	[109]
Head and neck squamous cell carcinoma (HNSCC)	PER2	miR-3187-3p	Promote HNSCC progression	[110]
**Cardiovascular Diseases (CVDs)**				
Atherosclerosis	BMAL1	lncRNA-AK023617	Promote atherosclerosis progression	[111]
	XAF1	miR-21	Promote atherosclerosis progression	[112]
	Clock and BMAL1	lnc NEAT1	Promote atherosclerosis progression	[113]
**Inflammation**				
Ulcerative colitis	BMAL1/ Rev-erbα	lnc-UC	Suppress ulcerative colitis	[114]
Steatohepatitis	Rev-erbα	lnc Platr4	Suppress steatohepatitis	
Sepsis	BMAL1	miR-155	Promote inflammation and sepsis progression	
**Others**				
Intervertebral disc degeneration (IDD)	BMAL1	hsa-let-7f-1-3p	Promote IDD progression	[117]
Age-related bone loss	BMAL1	miR-142-3p	Suppress bone formation	[118]

## Circulating non-coding RNAs (miRNAs or lncRNAs) as biomarkers in the diagnosis and prognosis of circadian disruption

LncRNAs and miRNAs have emerged as promising biomarkers for disease diagnosis and prognosis [119–121]. LncRNAs can be released into circulation and remain stable in blood, making them valuable for differentiating patients from the normal population [122]. These circulating ncRNAs demonstrate high abundance and stability, offering potential as non-invasive, blood-based biomarkers that provide information on disease biology and treatment effects [123]. Circulating lncRNAs can enter the circulatory system through exosomes, microvesicles, or in conjunction with RNA-binding proteins and are widely present in body fluids such as blood and urine [124].

**Fig. 4:**
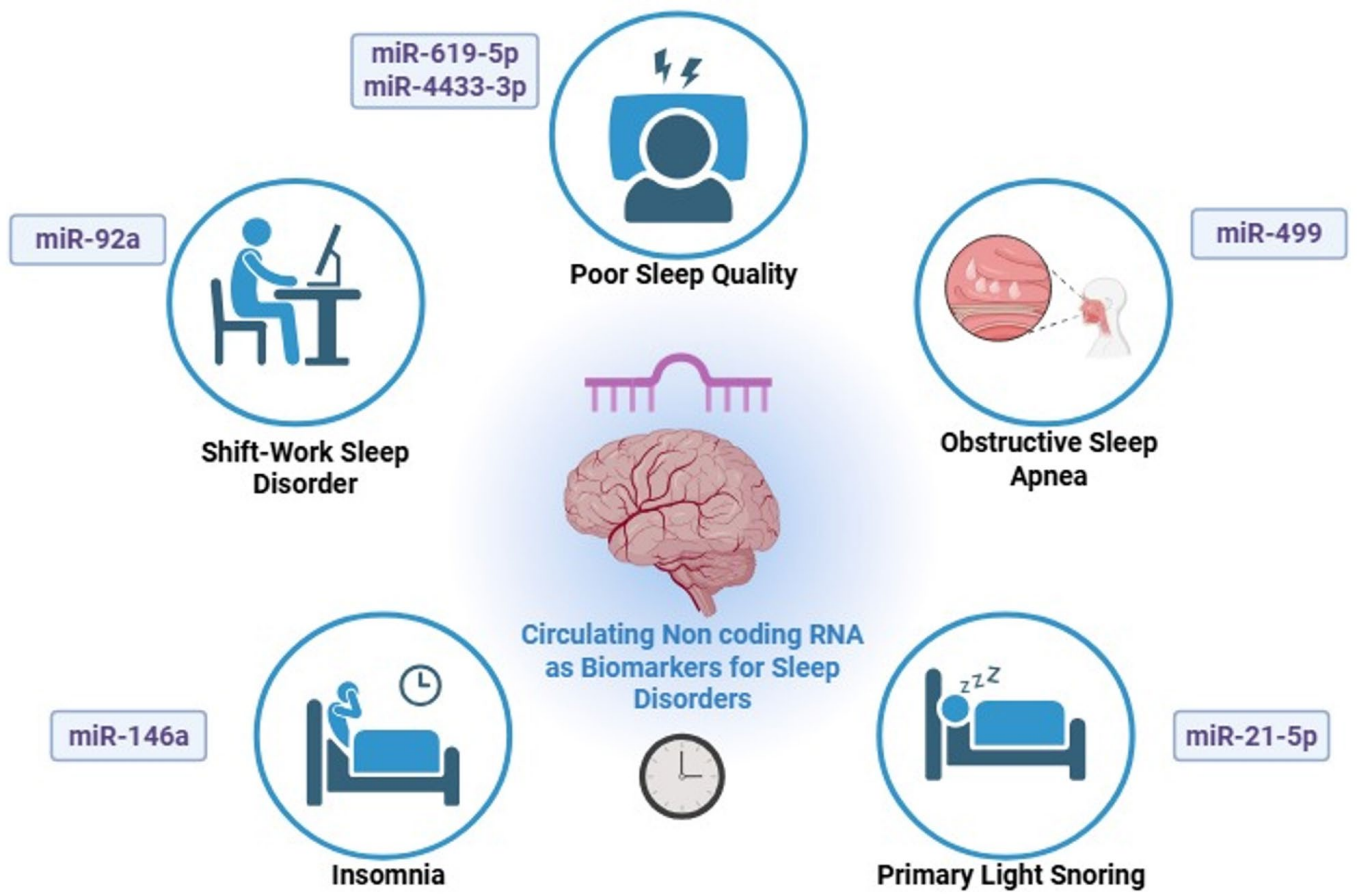
Circulating microRNAs associated with shift-work sleep disorder and other sleep-related conditions. Shift workers showed reduced serum exosomal miR-92a levels and increased brown adipose tissue compared with daytime workers. Further studies emphasize miRNA involvement in sleep disorders: combined miR-619-5p and miR-4433b-3p are suggested biomarkers of poor sleep quality; miR-499 is involved in hypoxia-related gene regulation in obstructive sleep apnea; and down-regulation of miR-21 and miR-23 with higher TNF-α suggests inflammatory pathways in OSA. Variants in miR-146a have also been associated with increased risk of fatal familial insomnia. **[**BAT, Brown Adipose Tissue; miRNA, MicroRNA; OSA, Obstructive Sleep Apnea; SNP, Single Nucleotide Polymorphism; TNF-α, Tumour Necrosis Factor-α.]

Shift work sleep disorder is a disorder that mostly affects those who work rotational, night, and early morning shifts. Bracci and his coworkers examined serum exosomes of miR-92a in 30 employees who worked shifts and throughout the day. While there was a variation in serum exosomal miR-92a levels between the two groups, there were no discernible metabolic changes between daytime and shift workers. The expression level of miR-92a was found to be decreased in shift workers, together with elevated brown adipose tissue (BAT) in comparison to daytime workers, as shown in Fig. 4 [125].

The pathophysiology of poor sleep quality has been linked to circulating miRNAs, suggesting that they may play a role in diagnosing sleep disorders. A study performed by Baek and his colleagues suggested that the combination of both miR-619-5p and miR-4433b-3p might be used as possible biomarkers for the diagnosis of poor sleep quality through modulating the circadian clock genes, which may be more useful than using either miRNA alone [126]. The most prevalent type of sleep-disordered breathing is obstructive sleep apnea (OSA), which is marked by the closing of the upper airways entirely or partially as you sleep, changing the flow of air. It was found that miR-499 may be a novel diagnostic marker for OSA and that it regulates the expression of genes during hypoxia [127]. Additionally, Chen et al. (2020) examined the anti-inflammatory activity of miR-21 and miR-23 through the TLR/TNF-α signaling pathway. When comparing OSA patients to those with primary light snoring, they found that TNF-α gene expression was elevated, and both miRNAs were down-regulated. These findings suggest that overexpressing miR-21-5p may be a novel treatment option for OSA in the future [128].

Insomnia, a common clinical condition marked by difficulty falling or staying asleep, with symptoms such as irritability or fatigue during wakefulness, is common in neurological diseases. Insomnia, excessive daytime sleepiness, or both are common symptoms of circadian rhythm sleep disorder [129]. An SNP in miR-146a has been linked to a higher risk of fatal familial insomnia, a prion disease that disrupts sleep and causes brain function to deteriorate and coordination to decline.

Despite the growing interest in circulating ncRNAs as biomarkers of circadian disruption, several methodological challenges currently limit their clinical translation. One major limitation is the pronounced circadian and diurnal variability of ncRNA expression, often referred to as “temporal noise,” whereby circulating ncRNA levels fluctuate according to circadian phase, sleep–wake cycles, feeding behaviour, hormonal rhythms, and environmental light exposure. Consequently, biomarker measurements obtained at different times of the day may yield substantial inter- and intra-individual variability, potentially reducing reproducibility and diagnostic accuracy. These challenges are particularly important in circadian medicine because ncRNA oscillations may reflect normal physiological rhythmicity rather than disease-specific alterations.

To improve the reliability and clinical applicability of circadian ncRNA biomarkers, future studies should incorporate standardized time-resolved sampling protocols. Factors including sampling time, fasting status, sleep timing, light exposure, medication use, and chronotype should be carefully controlled or recorded during study design. Repeated longitudinal sampling across multiple circadian phases may also be necessary to distinguish pathological circadian disruption from normal rhythmic fluctuations. Furthermore, harmonization of analytical methods, sample processing, and data normalization strategies will be essential to improve reproducibility across studies and clinical settings.

## The link between ncRNAs and circadian rhythm-personalized medicine approaches

The intersection of ncRNAs, circadian rhythm, and personalized medicine represents an emerging dimension of precision healthcare with important translational implications. From a circadian perspective, personalized medicine extends beyond classical pharmacogenomic variability and increasingly incorporates chrono-pharmacology, where the timing of drug administration influences therapeutic efficacy and toxicity. Circadian clocks regulate multiple pharmacokinetic and pharmacodynamic processes in a time-dependent manner, including drug absorption, hepatic metabolism, detoxification, renal elimination, transporter activity, and tissue responsiveness to therapy. Consequently, the efficacy and adverse effects of many drugs may vary according to circadian phase, forming the basis of chrono-pharmacokinetics and chronotherapy [130, 131].

Within this framework, ncRNAs introduce an additional layer of temporal regulation by modulating the rhythmic expression of clock genes and downstream drug-handling pathways. Several miRNAs have been reported to regulate drug-metabolizing enzymes, nuclear receptors, and transporters involved in absorption, distribution, metabolism, and excretion processes [132]. For example, miR-27b regulates CYP3A4 expression, whereas miR-328 has been implicated in controlling the drug transporter ABCG2, suggesting that ncRNA-mediated oscillations may influence both metabolic capacity and transporter-dependent drug disposition in a circadian manner [133, 134]. In parallel, core circadian regulators such as BMAL1 contribute to rhythmic expression of cytochrome P450 enzymes and detoxification pathways, further linking clock-controlled transcription with time-dependent pharmacological responses [132].

Importantly, these interactions may contribute to interindividual variability in chronopharmacokinetic and chronopharmacodynamic profiles. ncRNA-dependent regulation of clock-controlled pathways could alter drug sensitivity, therapeutic efficacy, and adverse-effect susceptibility across the day, particularly in disorders associated with circadian disruption such as cancer, metabolic diseases, inflammatory disorders, and sleep-related conditions. Accordingly, integrating ncRNA signatures with circadian phase biomarkers may improve chronotherapy design and support more precise timing of drug administration. Future personalized chrono-medicine approaches may therefore combine ncRNA profiling, circadian phenotyping, and time-resolved therapeutic scheduling to optimize treatment efficacy while minimizing toxicity [130–134].

Indeed, body homeostasis is regulated by circadian rhythms, which also affect metabolism and physiology. Several issues should be taken into account from the many 24-hour-omics datasets created in recent years, especially at this time when biomedical and translational research supports customized or precision treatment. Every individual is unique, and the same is true with circadian rhythms. It is important to evaluate inter-individual variability when considering clinical implementation, translational potential, and customized treatment. People may generally be divided into three basic chronotypes: those who like to go to bed and get up later, known as “night owls,” those who prefer to wake up early, sometimes known as “morning larks,” and those who fall somewhere in the middle. However, the picture is more complicated than that. We now understand that a variety of environmental influences, including exercise and food choices, can change rhythms particular to a given tissue. There are a number of known genetic mutations that cause a main circadian abnormality [135–137]. Secondary circadian disorders are simultaneous circadian abnormalities found in many other conditions. When it comes to treating circadian dysfunction, time is crucial; thus, phenotyping the circadian disruption before starting therapy is essential for both types of diseases. Since certain ailments are caused by a misalignment of endogenous rhythms, it is actually crucial to measure numerous rhythms within an individual [138]. Wearable technology for tracking activity and body location, as well as wrist and skin temperature monitoring, is a traditional technique for tracking the circadian phase and period in ambulatory individuals [139]. Furthermore, the chance to create new and possibly better biomarkers for determining an individual’s phase and period has been presented by the recent discovery that approximately half of the genome exhibits a rhythmic expression pattern. Lastly, mobile telephones offer a special and perhaps very successful way to get reliable information about a person’s circadian rhythms. For a circadian aberration to be successfully treated, it is therefore both technically possible and essential to gather solid data on a patient’s circadian phenotype [140, 141].

The combination of ncRNA regulations and circadian biology is one of the most exciting new areas in this research. By regulating the expression of key clock genes, including CLOCK, BMAL1, PER, and CRY, several ncRNAs-in particular, miRNAs, lncRNAs, and circRNAs have been demonstrated in earlier research to play crucial roles in preserving circadian rhythm. These ncRNAs can cause circadian misalignment through dysregulation, which can aid in the onset and progression of illness. This has made it possible to use ncRNA expression patterns as both therapeutic targets and biomarkers for circadian health. Crucially, ncRNAs also control transporters, receptors, and enzymes that break down drugs. This means that their expression can determine not only whether a drug is effective for a given patient, but also when it is best to take it to maximize effectiveness and minimize toxicity-a process known as chronotherapy [61, 142, 143]. It may be possible to enhance treatment results by arranging chemotherapy or miRNA-based medicines during particular circadian phases. Therefore, combining genetic profiling, ncRNA signatures, and chronotype evaluation might result in a very accurate, patient-specific, and time-sensitive treatment plan. By customizing the drug, dosage, and time of day, this paradigm is a potent advancement in precision medicine that will ultimately increase therapeutic efficacy, decrease side effects, and bring contemporary healthcare into harmony with the natural cycles of human biology.

## Challenges and future perspectives

Before being used in clinical practice, the integration of circadian rhythm and ncRNAs into personalized medicine has to conquer a number of significant obstacles. An important hurdle is the intricacy of circadian biology, which includes complicated feedback loops involving clock-regulated genes and ncRNAs that differ not just among persons but even within tissues within the same individual. Making a single “optimal” treatment duration which is successful for all organs or pharmacological targets is challenging as a result. Further, robust bioinformatics tools, portable devices, and established protocols for sampling at various time periods are necessary to capture accurate circadian rhythms since ncRNA profiling and chronobiological measures are not yet widespread in clinical settings.

Inter-individual variability also presents difficulties, such as genetic variants, lifestyle variables (such as nutrition, sleep habits, and light exposure), and comorbidities that can change ncRNA production and circadian rhythms, making therapy customization more difficult. An additional challenge in translating circadian ncRNA research into clinical practice is the issue of temporal variability in biomarker measurements. Because many circulating ncRNAs exhibit circadian oscillations, their expression profiles may vary considerably depending on sampling time and behavioural conditions. This temporal variability complicates comparisons across studies and may reduce reproducibility when sampling protocols are not standardized. Therefore, future clinical studies should incorporate time-resolved sampling frameworks and detailed circadian phenotyping to ensure accurate interpretation of ncRNA-based biomarkers and therapeutic responses. From a therapeutic perspective, problems with tissue selectivity, stability, and possible unwanted side effects continue to restrict the administration of ncRNA-based medications (such as miRNA mimics or inhibitors). Furthermore, there are logistical challenges: the implementation of chronotherapy necessitates careful planning, patient compliance, and collaboration across healthcare facilities.

The prospects for this field in the future appear very promising. Real-time integration of circadian data, ncRNA profiles, and pharmacogenomic information to create tailored therapy algorithms is anticipated to be made possible by developments in artificial intelligence and multi-omics technologies (genomics, transcriptomics, and epigenomics). It may soon be feasible to continuously monitor circadian indicators, including body temperature, heart rate variability, and hormone levels, using wearable biosensors and digital health platforms. This would enable therapeutic scheduling to be dynamically adjusted according to the patient’s present biological condition. Additionally, the development of evidence-based chrono-therapeutics is being facilitated by the growing number of clinical trials that are informed by chronobiology and evaluate the safety and effectiveness of drugs at various times of the day. With the use of ncRNA biomarkers and circadian profiling, doctors may provide the correct medication at the right time in the future, in addition to the appropriate dosage. Overall, integrating circadian biology with ncRNA profiling may support the development of more temporally informed and individualized therapeutic strategies.

## Conclusion

The rhythmic expression of core clock genes and the temporal control over physiological processes are shaped by ncRNAs, which are essential modulators of the circadian rhythm. Their imbalance disrupts the circadian balance, making people more susceptible to everything from cancer to cardiovascular and metabolic problems. In addition to offering insights into basic chronobiology, an understanding of the interaction between ncRNAs and circadian networks creates new possibilities for precision medicine. Specialists may soon be able to tailor medicine selection, dosage, and timing to each patient’s particular biological rhythm by using ncRNA-based biomarkers, treatments, and circadian profiling. To translate these findings, future work must concentrate on improving ncRNA delivery technologies, creating uniform time-resolved sampling techniques, and combining transcriptome and circadian data using AI-powered platforms. Finally, Chronotherapy and ncRNA biology together have the potential to revolutionize illness prevention and treatment by providing a more dynamic, time-sensitive, and individualized approach to medical care.

## Data Availability

No datasets were generated or analysed during the current study.

## Abbreviations

AE: Asparagus extract
AKT: Protein kinase B
ALDH: Aldehyde dehydrogenase
ADIRF-AS1: Adipogenesis regulatory factor antisense RNA 1
ARNTL: Aryl hydrocarbon receptor nuclear translocator-like 1
ARNTL2: Aryl hydrocarbon receptor nuclear translocator-like 2
Asc: Apoptosis-associated speck-like protein containing a CARD
BMAL1: Brain and muscle ARNT-like 1
BCSCs: Breast cancer stem-like cells
BMSCs: Bone marrow mesenchymal stem cells
Cbx1: Chromobox protein homolog 1
ceRNA: Competing endogenous RNA
circRNA: Circular RNA
CK1δ: Casein kinase 1 delta
CK1ε: Casein kinase 1 epsilon
CLOCK: Circadian locomotor output cycles kaput
CRY: Cryptochrome
CRY1/2: Cryptochrome 1/2
CVDs: Cardiovascular diseases
DARS2: Aspartyl-tRNA synthetase 2 mitochondrial
DGCR8: DiGeorge syndrome critical region gene 8
DNA: Deoxyribonucleic acid
EMT: Epithelial–mesenchymal transition
EZH2: Enhancer of zeste homolog 2
FBXL3: F-box and leucine-rich repeat protein 3
FBXL21: F-box and leucine-rich repeat protein 21
FLRL2: Fatty liver rhythmic lncRNA 2
frq: Frequency gene
GPAM: Glycerol-3-phosphate acyltransferase mitochondrial
H3K14ac: Histone H3 lysine 14 acetylation
H3K27me3: Histone H3 lysine 27 trimethylation
H3K4me3: Histone H3 lysine 4 trimethylation
HCC: Hepatocellular carcinoma
HIF-1α: Hypoxia-inducible factor 1 alpha
HNRNPA2B1: Heterogeneous nuclear ribonucleoprotein A2/B1
HNSCC: Head and neck squamous cell carcinoma
HULC: Highly upregulated in liver cancer
HUVECs: Human umbilical vein endothelial cells
IDD: Intervertebral disc degeneration
Irgm1: Immunity-related GTPase family M protein 1
lncRNA: Long non-coding RNA
LINC01234: Long intergenic non-protein coding RNA 1234
LPA: Lysophosphatidic acid
LPS: Lipopolysaccharide
MAPK: Mitogen-activated protein kinase
miRNA/miR: MicroRNA
MLL1: Mixed-lineage leukemia 1
mRNA: Messenger RNA
NAD+: Nicotinamide adenine dinucleotide
ncRNA: Non-coding RNA
NEAT1: Nuclear enriched abundant transcript 1
NF-κB: Nuclear factor kappa B
NLRP3: NOD-like receptor family pyrin domain containing 3
NPAS2: Neuronal PAS domain protein 2
NPCs: Nucleus pulposus cells
NR1D1: Nuclear receptor subfamily 1 group D member 1
NR1D2: Nuclear receptor subfamily 1 group D member 2
NRON: Non-coding repressor of NFAT
NSCLC: Non-small cell lung cancer
OsLAC: Oryza sativa laccase
OsPRR37: Oryza sativa pseudo-response regulator 37
PER: Period
PER1/2/3: Period circadian proteins 1/2/3
PER2AS: Period 2 antisense RNA
PI3K: Phosphatidylinositol 3-kinase
Platr4: Pluripotency-associated transcript 4
PTEN: Phosphatase and tensin homolog
PTMs: Post-translational modifications
qrf: Frequency antisense transcript
RAAS: Renin–angiotensin–aldosterone system
REV-ERBα/β: Reverse erythroblastosis virus alpha/beta
RIP3: Receptor-interacting protein kinase 3
RNA: Ribonucleic acid
RORα/β/γ: Retinoic acid receptor-related orphan receptor alpha/beta/gamma
ROREs: Retinoic acid-related orphan receptor response elements
RP11-414H17.5: Long non-coding RNA RP11-414H17.5
Rxrα: Retinoid X receptor alpha
SCN: Suprachiasmatic nucleus
SIRT1: Sirtuin 1
STEAP2-AS1: STEAP2 antisense RNA 1
SUMOylation: Small ubiquitin-like modifier conjugation
TIM: Timeless
TMAO: Trimethylamine N-oxide
TTFLs: Transcriptional–translational feedback loops
UCA1: Urothelial carcinoma associated 1
Wnt: Wingless-related integration site
XAF1: XIAP-associated factor 1
XIAP: X-linked inhibitor of apoptosis protein
YAP: Yes-associated protein
